# Surgery for hernia: quo vadis?

**DOI:** 10.4103/0972-9941.27719

**Published:** 2006-09

**Authors:** Tehemton E Udwadia

**Affiliations:** Head, Department of Minimal Access Surgery, P. D. Hinduja National Hospital, Veer Savarkar Marg, Mahim, Mumbai - 400016, India

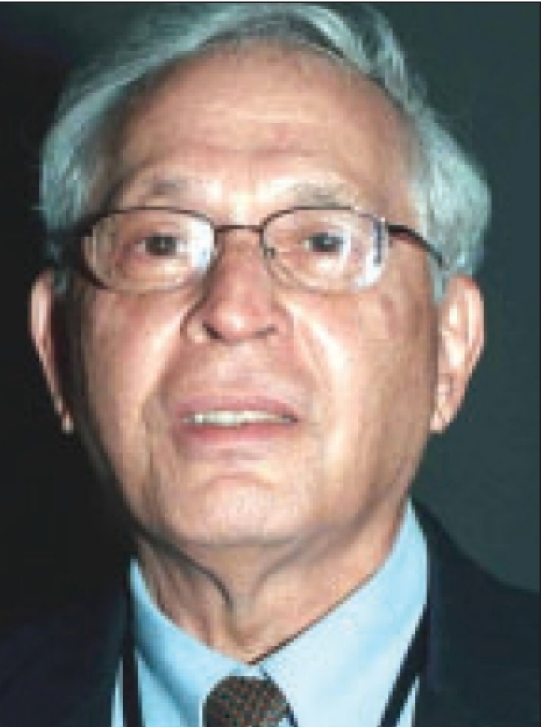



The Editorial Board of the Journal of Minimal Access Surgery wishes to extend their sincere gratitude to Dr. Pradeep Chowbey and his team from Department of Minimal Access Surgery, Sir Ganga Ram Hospital for taking on the onerous responsibility of bringing out this special issue dedicated to Hernia Surgery. They have worked tirelessly to meet some tough deadlines to ensure that the issue is released during the Second Congress of the Asia Pacific Hernia Society taking place in New Delhi. We also thank all the authors from around the world, who are all experts in the field of hernia surgery, for their informative and thought-provoking contributions.

The reader of a Minimal Access Surgery Journal may well question the publication of several articles unrelated to Minimal Access Surgery in this Journal. It is the view of this Editorial Board that surgery be seen and studied in its totality and in the current scenario, hernia represents the commonest surgical ailment which merits a wide-spectrum study. The average general surgeon who plods along without any pretensions of being a “herniologist”, nonetheless has repair of hernia as his commonest operative procedure. This issue of the JMAS is meant to address all surgeons, in all walks of life, on various facets of hernia repair.

The history of hernia is perhaps the most exciting and relevant aspect of surgical history and advance. From the early Greeks who coined the term hernia (Hernios = bud), through the ages of hernia treatment by cautery and castration, we have come a long way. Logical treatment for hernia commenced with an understanding of the anatomy and the establishment of sound surgical principles, a surgical treatment best described by Eduardo Bassini[1] in 1888 by his concept of tissue repair or rather tissue approximation. Bassini brought down the recurrence rate of hernia from 100 to 10%. This achievement is all the more creditable in a period with no antibiotics, no anaesthesia as we know it and the fact that all patients were in an advanced state of hernia progression. Though surgeons were aware of the problems of tissue repair, the Bassini procedure with several modifications was the mainstay of hernia repair for nine decades and for millions of patients till tension-free onlay mesh hernioplasty with various prosthetic materials, as standardized by Lichtenstein[2] ushered an era of less pain, shorter hospitalization, lower recurrence and early return to work after hernia surgery.

With the advent of laparoscopy entering every field of surgery, laparoscopic hernia repair was the obvious next step. For several reasons, unlike laparoscopic cholecystectomy, which had a market penetration of 93% within 3 years, laparoscopic hernia repair after 16 years has enjoyed or rather suffered, a market penetration of 5–15% in the developed world.[3–5] However the contribution of laparoscopic hernia repair goes way beyond its poor acceptance. Hitherto, in spite of or perhaps because of its common occurrence, hernia repair was relegated to a minor, almost step-child, position in surgery. Laparoscopic hernia repair has focused world surgical attention on the treatment of hernia and elevated it to its current status of study, controversy, evaluation and treatment.

Papers at conferences and articles in journals on hernia repair are by and large contributed by “Herniologists” who work and practice in ideal conditions as they apply in developed countries or in urban centers of excellence in developing countries. However, the vast mass of patients in the Asia- Pacific region does not come under the gambit of “centers of excellence”. If it is our intent to go beyond mere lip-service to the vast population of hernia patients, we need to rethink our strategies in terms of economics and management to enable surgeons to offer the best possible treatment to all patients in all places, irrespective of national or economic barriers.
